# Ultrafine carbon particle mediated cardiovascular impairment of aged spontaneously hypertensive rats

**DOI:** 10.1186/s12989-014-0036-6

**Published:** 2014-09-17

**Authors:** Swapna Upadhyay, Tobias Stoeger, Leema George, Mette C Schladweiler, Urmila Kodavanti, Koustav Ganguly, Holger Schulz

**Affiliations:** Institute of Lung Biology and Disease, Comprehensive Pneumology Center Helmholtz Zentrum München, German Research Center for Environmental Health, Munich, Germany; Department of Biotechnology, Indian Institute of Technology, Madras, Chennai, 600036 India; SRM Research Institute, SRM University, Chennai, 603203 India; Environmental Public Health Division, National Health and Environmental Effects Research Laboratory, U.S. Environmental Protection Agency, Research Triangle Park, North Carolina, NC 27711 USA; Institute of Epidemiology I, Helmholtz Zentrum München, German Research Center for Environmental Health, D-85764 Neuherberg/München, Germany

**Keywords:** Inflammation, Lung, Heart, Dust, Particle exposure, Cardiovascular risk individuals

## Abstract

**Background:**

Studies provide compelling evidences for particulate matter (PM) associated cardiovascular health effects. Elderly individuals, particularly those with preexisting conditions like hypertension are regarded to be vulnerable. Experimental data are warranted to reveal the molecular pathomechanism of PM related cardiovascular impairments among aged/predisposed individuals. Thus we investigated the cardiovascular effects of ultrafine carbon particles (UfCP) on aged (12–13 months) spontaneously hypertensive rats (SHRs) and compared the findings with our pervious study on adult SHRs (6–7 months) to identify age related predisposition events in cardiovascular compromised elderly individuals.

**Methods:**

Aged SHRs were inhalation exposed to UfCP for 24 h (~180 μg/m^3^) followed by radio-telemetric assessment for blood pressure (BP) and heart rate (HR). Bronchoalveolar lavage (BAL) fluid cell differentials, interleukin 6 (IL-6) and other proinflammatory cytokines; serum C-reactive protein (CRP) and haptoglobin (HPT); and plasma fibrinogen were measured. Transcript levels of hemeoxygenase 1 (HO-1), endothelin 1 (ET1), endothelin receptors A, B (ETA, ETB), tissue factor (TF), and plasminogen activator inhibitor-1 (PAI-1) were measured in the lung and heart to assess oxidative stress, endothelial dysfunction and coagulation cascade.

**Result:**

UfCP exposed aged SHRs exhibited increased BP (4.4%) and HR (6.3%) on 1^st^ recovery day paralleled by a 58% increase of neutrophils and 25% increase of IL-6 in the BAL fluid. Simultaneously higher CRP, HPT and fibrinogen levels in exposed SHRs indicate systemic inflammation. HO-1, ET1, ET-A, ET-B, TF and PAI-1 were induced by 1.5-2.0 folds in lungs of aged SHRs on 1^st^ recovery day. However, in UfCP exposed adult SHRs these markers were up-regulated (2.5-6 fold) on 3^rd^ recovery day in lung without detectable pulmonary/systemic inflammation.

**Conclusions:**

The UfCP induced pulmonary and systemic inflammation in aged SHRs is associated with oxidative stress, endothelial dysfunction and disturbed coagulatory hemostasis. UfCP exposure increased BP and HR in aged SHRs rats which was associated with lung inflammation, and increased expression of inflammatory, vasoconstriction and coagulation markers as well as systemic changes in biomarkers of thrombosis in aged SHRs. Our study provides further evidence for potential molecular mechanisms explaining the increased risk of particle mediated cardiac health effects in cardiovascular compromised elderly individuals.

## Introduction

Air pollution mediated cardiopulmonary effects are one of the major public health concerns in industrialized cities throughout the world [1-4]. It is well known that episodes of air pollution are associated with increased emergency room visits due to cardiopulmonary impairments as well as increased morbidity and mortality rates. The majority of the effects occurred among elderly individuals and those with pre-existing disorder/s like cardiovascular diseases (CVD), respiratory ailments and diabetes [5,6]. There is a growing concern about the potential health hazards caused particularly by increased emissions of combustion derived ultrafine particles (UFPs) from motor vehicles [7,8]. Motor-vehicle emissions consist of a complex mixture of particulate, chemical and gaseous pollutants such as fine particulate matter (PM2.5; diameter < 2.5 μm), ultrafine particles (UFPs; diameter < 0.1 μm), metals, volatile organic material, black carbon, ozone etc. [7,8]. Several epidemiological studies suggested that the carbonaceous UFPs emitted from diesel-powered motor vehicles and other combustion sources induce airway inflammation and reduced lung function [9,10]. Nemmar et al. [11] reported that inhalation of ambient ultrafine carbon particles (UfCP) and/or nanoparticles not only induce inflammatory responses and oxidative stress but may also have immune-suppressive effects, impairing macrophage function and altering epithelial barrier functions. Furthermore, the initiated inflammatory reaction releases mediators capable of exacerbating lung disease and increasing blood coagulability among predisposed individuals [12]. Similarly Schwarze et al. [13] have shown series of evidence suggesting that prolonged airway inflammation associated with PM exposure may lead to systemic inflammation which may increase the susceptibility to acute cardiovascular effects. UfCP, by virtue of their small size can easily penetrate into the deep lung (alveolar compartment) and also impair airway mucocillary clearance [14,15]. UfCP and components of PM can enter into the blood stream and are translocated to extrapulmonary organs like heart, liver, brain [16-18]. The large surface area of UfCP facilitates release of soluble potentially toxic chemicals thereby causing cell injuries [19-22]. UfCP constitutes the core of combustion derived particles and represents relevant surrogates for exhaust particles from modern diesel engines [23-25]. UfCPs regardless of their different sources like combustion-derived ultrafine particles or carbon based engineered nanoparticles, are often included in commonly applied products such as rubber, inks, paints and reinforcing agents in tires are of toxicological interest given their small dimensions with properties not displayed by their macroscopic counterparts [26,27]. The pulmonary deposition efficiency of inhaled UFPs, along with chemical constituents easily absorbed on their large surface area, are considered to drive the emerging health effects linked to cardiopulmonary toxicity. In this study we used well characterized UfCPs containing >96% elemental carbon [28] as a surrogate to address the direct toxicity of the carbon particle core without contributing effects of organic compounds or metals.

Scientific statements of American Heart Association [29] as well as extensive review by Gold and Mittleman [30] discussed in detail the higher risk for cardiovascular mortality due to PM exposure for few hours to weeks. There is ample evidence from epidemiological studies showing the elevated risk of myocardial infarction (MI), stroke, arrhythmia, reduced heart rate variability (HRV), and increased blood pressure (BP) following exposure in susceptible individuals [29]. Gold and Mittleman [30] and Nemmar et al. [11] have further described several plausible pathological mechanisms of pollutant mediated CVDs. These includes pulmonary and/systemic inflammation, oxidative stress, alteration in prothrombotic and coagulation pathways, vasoconstriction and altered BP, vascular dysfunction and progression of atherosclerosis. Researchers have also correlated variety of health effects, particularly respiratory and cardiovascular impairments, in response to the size and/or chemical composition of PM and population characteristics (age, genetic predisposition, pre-existing conditions). However, the underlying pathophysiological features which predispose an individual to cardiovascular impairments following exposure to air pollutants such as PM are still not well elucidated.

Previously we detected that 24 h inhalation exposure of UfCP serving as surrogates for combustion derived UfCP in adult spontaneously hypertensive rats (age: 6 months; SHRs) cause cardiovascular impairments without detectable pulmonary inflammation [31]. Based on reported epidemiological observations and our previous findings we hypothesized that cardiovascular compromised elderly individuals are more susceptible and are at higher risk to cardiovascular impairments following UfCP exposure compared to younger compromised or healthy individuals. Thus, in this study we investigated the cardiovascular effects on aged SHRs (12 months) following UfCP exposure under similar conditions as previously described [31]. Furthermore, we compared the findings to our previous findings so as to identify the potentially susceptibility attributes (age/preexisting complications/higher disease burden) that can lead to increased risk of UfCP mediated health effect. Using comparable UfCP concentration, we detected significant pulmonary neutrophilic influx along with cytokine release in aged SHRs, which might be responsible for ultimate exacerbation of the observed cardiovascular impairments.

## Results

Aged SHRs served as their own control and were exposed first to filtered air and 4 weeks later to UfCP for 24 h to assess the effect on cardiac performance using a radio telemetric system. The 4 weeks time gap between control and exposure experiments was chosen to ensure elimination of any possible effect of clean air exposure on SHRs. Cardiac effects were most pronounced on the 1^st^ and 2^nd^ recovery day. CVD related biomarkers were further analyzed at the target site (lung) and systematically (heart and blood) in animals without telemetric implant on the 1^st^ and 3^rd^ recovery days following 24 h exposure to UfCP to assess inflammation, oxidative stress, endothelial dysfunction, and coagulation cascade. Since no significant alterations were observed on 3^rd^ recovery day, all data presented are from the 1^st^ recovery day.

### Cardiophysiological response in aged SHRs assessed by radio telemetry

#### Baseline conditions

**Figure 1:**
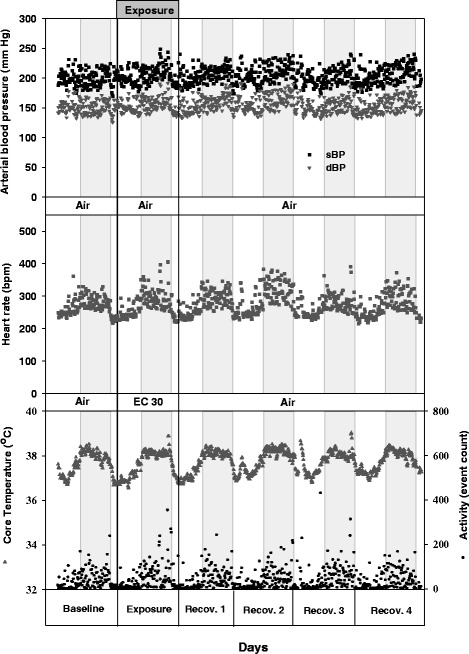
**Circadian rhythmicity of physiological and behavioral parameters of spontaneously hypertensive rats (SHRs).** The figure shows representative diastolic blood pressure (dBP), systolic blood pressure (sBP), heart rate (HR), body temperature (T) and physical activity (Act) during baseline, exposure and recovery periods of a single SHR rat. The gray segments indicate the dark periods (night time). Each data point of every parameter represents an average of 10-minutes data segments.

**Figure 2:**
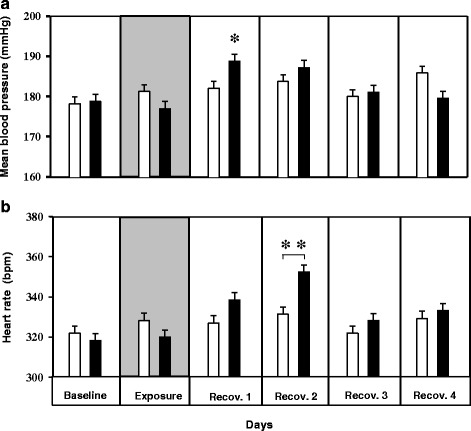
**Blood pressure (BP) and heart rate (HR) changes in spontaneously hypertensive rats (SHRs) following ultrafine carbon particle (UfCP) exposure compared to control. a**. Mean blood pressure (mBP) increased by 8 mmHg (4.4%) on the 1^st^ recovery day in the UfCP exposed SHRs compared to control. **b**. HR responded with a lag of one day and exhibited an increase on the 2^nd^ recovery day by 21 bpm (6.3%) in UfCP exposed SHRs compared to control. It reached baseline values on the 4^th^ recovery day. The vertical bars represent arithmetic mean values (mean ± SE) of control (white) and exposed (black) groups. Each bar represents a combined mean value of: (72 10-minutes segments/12 h dark periods/rat) × 7 rats. (n = 7/experimental group; *indicates p < 0.05).

Systolic and diastolic arterial blood pressure (sBP and dBP respectively), HR, body temperature (T), and activity level (Act) of a representative aged SHR for the time course of baseline (day 0), exposure (day 1), and recovery (day 2–5) periods at 10-minutes data segments are shown in Figure 1. Similar responses have been also observed among all other animals. The data reflects typical circadian rhythmicity of physiological and behavioural (activity) parameters of aged SHRs characterized by elevated BP, HR, T, and Act values during the dark periods. The findings are in accordance to our previous observations in adult SHRs [31]. Comparison of baseline values of cardiovascular parameters (mBP, HR, T, Act,) before clean air or UFP exposure in aged SHRs revealed no significant alterations despite the 4 week time gap (Figures 1, 2a and b; Table 1).

**Table 1:** **Baseline cardiovascular parameters of aged spontaneously hypertensive rats (SHRs, n = 7) in 4 weeks time gap before clean air exposure (12 months) or Ultrafine carbon particle (UfCP)-exposure (13 months)**

**Cardiovascular parameters**	**Baseline clean air exposure (12 months)**	**Baseline UfCP-exposure (13 months)**
Mean blood pressure (mBP, mmHg)	177 ± 1.8	179 ± 1.7
Heart rate (HR, bpm)	322 ± 3.7	317 ± 3.1
Body temperature (T, °C)	38.1 ± 0.1	37.9 ± 0.2
Activity (Act, event count)	54.1 ± 2.5	53.8 ± 4.1

#### Cardiovascular response to UfCP exposure

mBP were increased by 8 mmHg i.e. ~4.4% (p < 0.05) in UfCP exposed aged SHRs (control: 181 ± 1.5; exposed: 189 ± 1.8 mmHg) on the 1^st^ day of recovery and returned to baseline levels on the 3^rd^ day of recovery (Figure 2a). This significant increase of mBP (p < 0.05) is due to the concurrent increase in sBP (control: 208 ± 2.0; exposed: 213 ± 1.7 mmHg) and dBP (control: 154 ± 2.1; exposed: 155 ± 3.0 mmHg) with the effect on sBP being more pronounced.

In contrast to the response in BP, HR responded with a lag of one day similar to our previous observation in adult SHRs [31]. However significant increase of HR was detected only on the 2nd day of recovery. HR increased by 21 bpm (p < 0.05) i.e. ~6.3% in exposed SHRs (control: 331 ± 3.2; exposure: 352 ± 3.4 bpm) and reached baseline level on the 3^rd^ day of recovery (Figure 2b). UfCP exposure did not affect body temperature or activity level of the animals.

**Figure 3:**
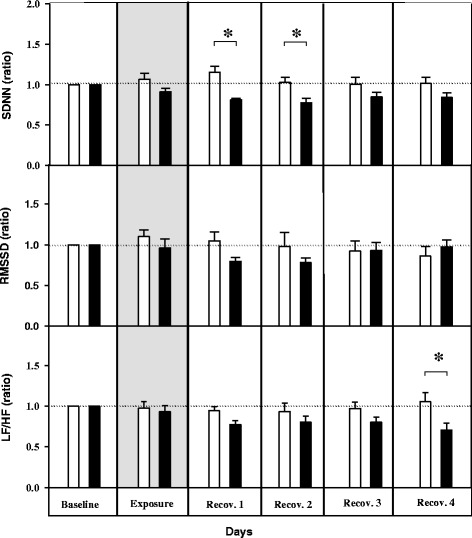
**Relative changes in time domain and frequency domain measurements of heart rate variability (HRV) in spontaneously hypertensive rats (SHRs) following ultrafine carbon particle (UfCP** **) exposure compared to control.** HRV paralleled changes in heart rate (HR). HRV was decreased by ~36% in UfCP exposed SHRs during the 1^st^ and 2^nd^ days of recovery period as indicated by altered SDNN. Dotted horizontal lines are used to highlight the relative changes. Each bar represents a combined mean value of: (12 5-minutes segments/12 h dark period/rat) × 7 rats. (n = 7/experimental group; *indicates p < 0.05). **SDNN:** standard deviation of normal to normal (NN) intervals. **RMSSD:** square root of the mean of squared differences between adjacent NN intervals. **LF/HF:** ratio of the absolute powers in the low-frequency (LF: 0.20 Hz to 0.75 Hz) and high-frequency bands HF: 0.75 Hz to 2.5 Hz).

Standard deviation of normal adjacent sinus intervals (SDNN), a measure of the overall HRV was decreased by ~36% during the 1^st^ (control: 1.15 ± 0.07; exposure: 0.81 ± 0.01 unit) and 2^nd^ (control: 1.03 ± 0.06; exposure: 0.77 ± 0.05 unit) recovery days (Figure 3). The square root of the mean of squared differences between adjacent normal to normal intervals (RMSSD) and the low-frequency to high-frequency ratio (LF/HF) showed a comparable response as SDNN but reached statistical significance only at the 4^th^ day of recovery (Figure 3). Similar response has been observed previously in UfCP exposed adult SHRs. No individual changes in absolute LF (baseline: 20.0 ± 2.0 nu; 1^st^ recovery day: 16.7 ± 3.0 nu; 2^nd^ recovery day: 16.9 ± 3.3 nu) and HF (baseline: 68.2 ± 4.9 nu; 1^st^ recovery day: 70.0 ± 6.8 nu; 2^nd^ recovery day: 67.0 ± 7.3 nu) were observed. However, a significant decrease in LF/HF ratio by 29% was observed in exposed SHRs on the fourth recovery day (control: 1.0 ± 0.1; exposure: 0.71 ± 0.08 unit, (p < 0.05). The observed transient increase in HR associated with overall decrease in HRV (i.e. SDNN) suggests an altered sympatho-vagal balance in response to UfCP exposure in aged SHRs.

### Pulmonary inflammatory response

#### Bronchoalveolar lavage

**Figure 4:**
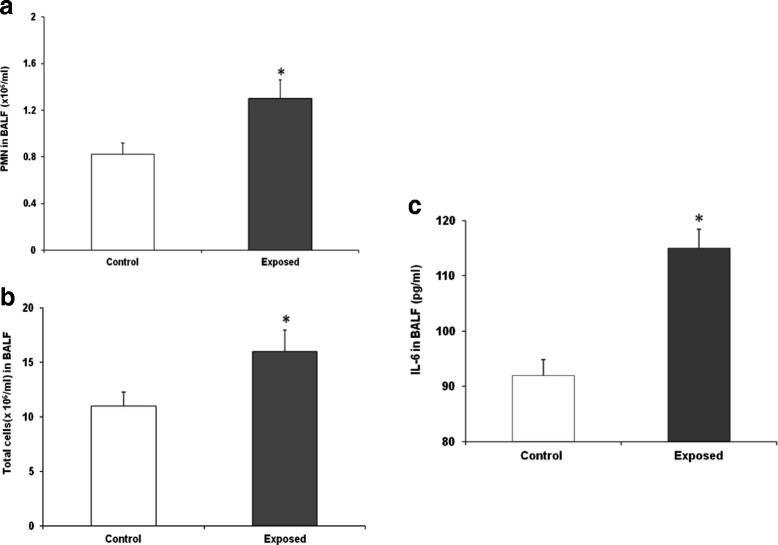
**Bronchoalveolar lavage (BAL) fluid cell differentials and interleukin 6 (IL6) concentrations in ultrafine carbon particle (UfCP** **) exposed spontaneously hypertensive rats (SHRs) compared to control on 1** ^**st**^ **recovery day. a**. Total cell numbers in the BAL fluid of UfCP exposed SHRs was increased by 41% compared to control (control: 11.6 ± 1.3; exposed: 16.4 ± 2.2 1/ml × 10^6^ cells, p < 0.05). **b**. Polymorphonuclear neutrophils (PMN) in the BAL fluid of UfCP exposed SHRs also increased by 58% compared to control (control: 0.82 ± 0.1; exposed: 1.3 ± 0.2 1/ml × 10^6^ cells, p < 0.05). **c**. IL6 concentration in BAL fluid of UfCP exposed SHRs increased by 25% compared to control. Data are represented as mean ± SE. (n = 6/experimental group; *indicates p < 0.05; white bar: control; black bar: exposed).

(BAL) fluid of aged SHRs was assessed on 1^st^ and 3^rd^ days of recovery following 24 h UfCP exposure based on the observed alterations in cardiac performance. Pulmonary inflammation has been observed only on the 1^st^ recovery day in UfCP exposed aged SHRs (Figure 4a and b). Total BAL cell count increased by 41% (control: 11.6 ± 1.3; exposed: 16.4 ± 2.2 1/ml × 10^6^ cells, p < 0.05) and polymorphonuclear neutrophils by 58% (PMN control: 0.82 ± 0.1; exposed: 1.3 ± 0.2 1/ml × 10^6^ cells, p < 0.05) in the BAL fluid of UfCP exposed SHRs compared to control while lymphocytes remained unchanged (Table 2). The levels of the proinflammatory cytokine interleukin 6 (IL6, Figure 4c) was increased by 25% in UfCP exposed aged SHRs on the 1^st^ recovery day (control: 92 ± 2.8; exposed: 115 ± 3.4 pg/ml, p < 0.05). Tumor necrosis factor α (TNFα) levels as well as parameters indicating tissue integrity like total protein, albumin concentration, γ-Glutamyltransferase (GGT), N-acetyl glucosaminidase (NAG), assessed in the BAL fluid did not show any change on 1^st^ and 3^rd^ recovery days in UfCP exposed SHRs compared to control. Transcript levels of TNFα and macrophage inflammatory protein 2 (MIP2) were also not altered in the lung tissue.

**Table 2:** **Comparison of ultrafine carbon particle (UfCP) induced cardiovascular and inflammatory effects and alterations of hematological parameters**

**Matrix**	**Adult SHRs (Age: 6–7 months) (Upadhyay et al.** **[** 25 **]**		**Aged SHRs (Age: 12–13 months)**	
	**1** ^**st**^ **-3** ^**rd**^ **recovery day**		**1** ^**st**^ **recovery day**	
**Cardiovascular**	**Control**	**UfCP exposed**	**Control**	**UfCP exposed**
BP (mmHg)	176 ± 2.0	182 ± 2.1 ↑ (3.4%)	181 ± 1.5	189 ± 1.8 ↑ (4.4%)
HR (bpm)	328 ± 3.5	345 ± 3.5 ↑ (5.2%)	331 ± 3.2	352 ± 3.4 ↑ (6.3%)
SDNN	1.08 ± 0.05	0.77 ± 0.03 ↓ (30%)	1.15 ± 0.07	0.81 ± 0.01 ↓ (36%)
**BAL**				
Total Cells (×10^6^/ml)	3.7 ± 0.2	4.7 ± 0.2	11.6 ± 1.3	16.4 ± 2.21 ↑ (41%)
PMN (×10^6^/ml)	0.3 ± 0.1	0.4 ± 0.1	0.8 ± 0.1	1.3 ± 0.2 ↑ (58%)
IL-6 (pg/ml),	83 ± 6.2	92 ± 4.1	92 ± 2.8	115 ± 3.4 ↑ (25%)
TNF-alpha (pg/ml)	Not detectable	Not detectable	20.0 ± 2.0	24.4 ± 2.6
NAG	5.3 ± 0.1	5.8 ± 0.1	4.5 ± 0.9	3.5 ± 0.2
**Hematology**				
WBC (^.^10^3^ cells/μl)	6.19 ± 0.4	5.79 ± 0.5	5.27 ± 0.7	5.57 ± 0.21
PLT (10^3^ cells/μl)	436 ± 30.2	443.8 ± 48.4	379.5 ± 50.1	456.7 ± 25.6 ↑ (20%)
Hematocrit	44.8 ± 0.8	43.8 ± 2.1	43.6 ± 1.4	44.1 ± 0.4
Thrombocrit (%)	0.51 ± 0.04	0.39 ± 0.05	0.38 ± 0.04	0.42 ± 0.02
Neutrophil (%)	48.9 ± 2.2	39.2 ± 2.8	49.8 ± 4.04	43.9 ± 1.5
Lymphocyte (%)	43.3 ± 3.1	56.3 ± 2.9	43.4 ± 4.4	49.3 ± 1.7
**Systemic**				
HPT (serum, mg/dl)	300 ± 17	309 ± 20	611 ± 17	710 ± 20 ↑ (16%)
CRP (serum, μg/ml)	92 ± 3.8	94 ± 3.0	132.7 ± 10.2	209 ± 4.4 ↑ (58%)
Fibrinogen (Plasma, mg/dl)	190 ± 11.4	198 ± 5.2	191 ± 5	217 ± 8.0 ↑ (13.6%)
Plasma renin concentration (PRC)	**1** ^**st**^ **recovery day**	**2** ^**nd**^ **recovery day**		
	55 ± 7.0/120 ± 14.0 ↑	52 ± 4.0/70 ± 8.0 ↑	50 ± 13.4	39 ± 9
**Lung Transcript analysis** (Expression in UfCP exposed SHRs compared to control)	**1** ^**st**^ **recovery day**	**3** ^**rd**^ **recovery day**	**1** ^**st**^ **recovery day**	
HO-1	NS	↑2.5 fold	↑2.0 fold	
ET-1	NS	↑6.0 fold	↑1.5 fold	
ET-A	NS	↑2.5 fold	↑1.7 fold	
ET-B	NS	↑2.5 fold	NS↓0.5 fold	
PAI-1	↓	↑2.5 fold	NS↓0.4 fold (3^rd^ day)	
TF	NS	↑2.5 fold	↑1.7 fold	
**Cardiac Transcript analysis** (Expression in UfCP exposed SHRs compared to control)	**1** ^**st**^ **recovery day**	**3** ^**rd**^ **recovery day**	**1** ^**st**^ **recovery day**	
HO-1	NS	↓2.0 fold	NS	
ET-1	NS	NS	↑1.5 fold	
ET-A	NS	NS	NS	
ET-B	NS	NS	NS	
PAI-1	NS	NS	NS	
TF	NS	NS	NS	

**Parameters are displayed for adult (6–7 months [** 25 **]) and aged (12–13 months) spontaneously hypertensive rats (SHRs)** Adult SHRs data are adapted from Upadhyay et al. [31]. **WBC**: White Blood Cells; **PLT**: Total Platelet; **BP**: blood pressure; **HR**: heart rate; **BAL**: broncho alveolar lavage; **IL6**: interleukin 6; TNF: tumor necrosis factor alpha; **NAG**: N-acetyl glucosaminidase; **HPT**: haptoglobin**; CRP**: C reactive protein. NS not significant. **↑**: significantly increased; **↓**: significantly decreased; Adult SHRs UfCP mediated effect was mostly observed with a lag of 1–3 days, whereas UfCP mediated immediate effect was observed on aged SHRs.

### UfCP mediated direct effect on pulmonary and cardiac tissue

#### Histopathology

Histopathological analysis of the lung and heart revealed no changes due to UfCP exposure in aged SHR rats compared to control (data not shown). Fibrotic foci (typical for SHRs) were noted in the heart but no indications of exposure related inflammatory reaction or cardiomyopathy was observed.

#### Lung transcript analysis

**Figure 5:**
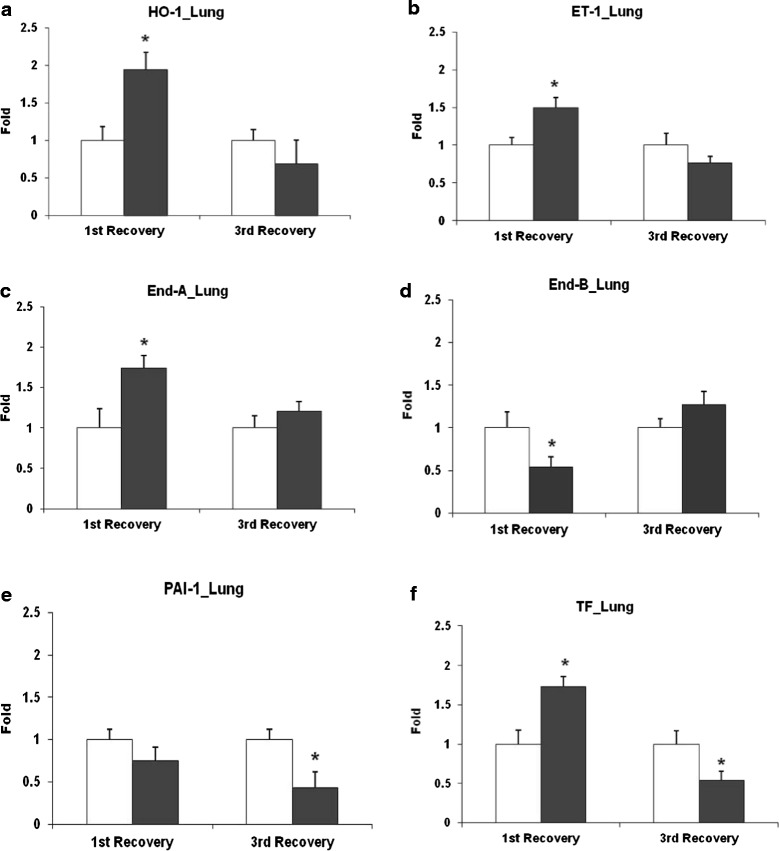
**Expression analysis of lung transcripts associated with oxidative stress, endothelial activation and coagulation cascade in ultrafine carbon particle (** **UfCP** **) exposed spontaneously hypertensive rats (SHRs) compared to control on 1** ^**st**^ **(24 h) and 3** ^**rd**^ **(72 h) recovery day using quantitative real time polymerase chain reaction (qRTPCR). a**. Transcript levels of Haemoygenase 1 (HO1) was significantly increased in UfCP exposed SHRs on 1^st^ recovery day compared to control whereas at 3^rd^ recovery day it remained unchanged. **b**. Transcript levels of endothelin 1 (ET1), was significantly increased in exposed SHRs on 1^st^ recovery day compared to control whereas at 3^rd^ recovery day it remained unchanged. **c**. Transcript levels of endothelin receptor A (ETA) was significantly increased in UfCP exposed SHRs on 1^st^ recovery day compared to control whereas at third recovery day it remained unchanged. **d**. Transcript levels of endothelin receptor B (ETB) was significantly reduced in UfCP exposed SHRs on 1^st^ recovery day compared to control whereas at 3^rd^ recovery day it remained unchanged. **e**. Transcript levels of plasminogen activator inhibitor-1 (PAI1) were significantly reduced in UfCP exposed SHRs on both 1^st^ and 3^rd^ recovery days compared to control. **f**. Transcript levels of tissue factor (TF) were significantly increased on 1^st^ recovery day and significantly reduced on 3^rd^ recovery day in UfCP exposed SHRs compared to control. Data are represented as mean ± SE. (n = 6/experimental group; *indicates p < 0.05; white bar: control; black bar: exposed.

Transcript profiling of markers associated with oxidative stress (hemeoxygenase 1: HO1), endothelial activation (endothelin 1: ET1; endothelin receptor A and B: ETA and ETB), and coagulation cascade (tissue factor: TF and plasminogen activator inhibitor-1: PAI-1) were assessed in the lungs of UfCP exposed aged SHRs compared to control on 1^st^ and 3^rd^ recovery day (Figure 5a, b, c, d, e and f) using quantitative real time polymerase chain reaction (qRT-PCR). Transcripts of HO1 (2.0 fold), ET1 (1.5 fold) and ETA (1.7 fold) were significantly (p < 0.05) increased in UfCP exposed aged SHRs on 1^st^ recovery day compared to control whereas at 3^rd^ recovery day HO-1 expression almost returned to control levels. Transcripts of ETB were reduced in UfCP exposed SHRs on 1^st^ recovery day by 0.5 fold (p < 0.05) whereas on 3^rd^ recovery day it remained unchanged. In contrast, transcripts of PAI1 were significantly reduced (0.4 fold, p < 0.05) in UfCP exposed SHRs on 3^rd^ recovery days compared to control. TF was increased on 1^st^ recovery day (1.7 fold, p < 0.05) but reduced (0.5 fold, p < 0.05) on 3^rd^ recovery day in UfCP exposed SHRs compared to control (Table 2).

#### Cardiac transcript analysis

**Figure 6:**
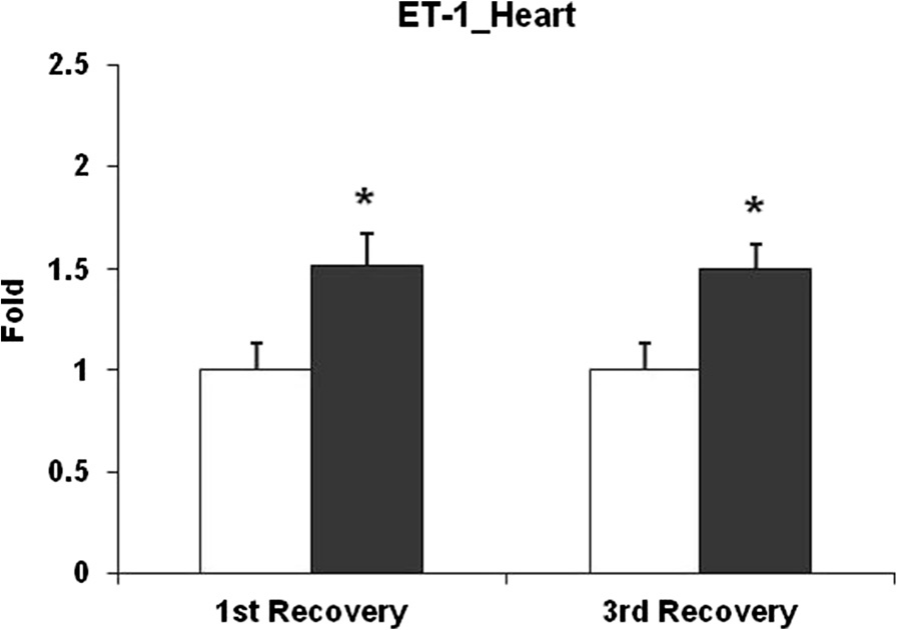
**Expression analysis of endothelin 1 (ET1) heart transcript level in ultrafine black carbon particle (UfCP) exposed spontaneously hypertensive rats (SHRs) compared to control on 1** ^**st**^ **(24 h) and 3** ^**rd**^ **(72 h) recovery day using quantitative real time polymerase chain reaction (qRTPCR).** Transcript levels of ET1 were significantly increased in UfCP exposed SHRs on both 1^st^ and 3^rd^ recovery days compared to control. Data are represented as mean ± SE. (n = 6/experimental group; *indicates p < 0.05; white bar: control; black bar: exposed).

Transcript expression of HO1, ET1, ETA, ETB, PAI1 and TF were also measured in the heart of UfCP exposed SHRs on the 1st and 3^rd^ day of recovery compared to control. Only ET1 was induced by 1.5 fold (p < 0.05) in the cardiac tissue of UfCP exposed SHRs on both 1^st^ and 3^rd^ recovery days (Figure 6; Table 2).

### Systemic response

#### Haematology

The complete blood cell count (total red and white blood cell, haematocrit, platelets, PMN, and lymphocytes) were measured in UfCP exposed aged SHRs on 1^st^ and 3^rd^ days of recovery compared to control (Table 2). Platelet numbers were significantly increased by 20% in UfCP exposed SHRs compared to control (control: 380 ± 50; exposed: 457 ± 25 ×10^3^ cells/μl, p < 0.05) on the 1^st^ day of recovery. Other cell counts remained unaffected.

#### Acute phase proteins

**Figure 7:**
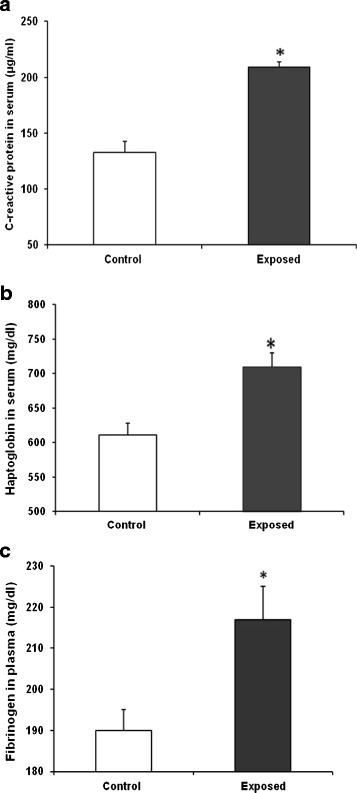
**Expression of acute phase protein in serum and plasma of ultrafine carbon particle (** **UfCP** **) exposed spontaneously hypertensive rats (SHRs) compared to control on 1** ^**st**^ **recovery day. a**. C-reactive protein (CRP) concentration increased by 58% (~1.6 fold) in the serum of UfCP exposed SHRs compared to control. **b**. Haptoglobin concentration in the serum of UfCP exposed SHRs after increased by 16% compared to control. **c**. Fibrinogen concentration in plasma of UfCP exposed SHRs increased by 13.6%. Data are represented as mean ± SE. (n = 8/experimental group; *indicates p < 0.05; white bar: control; black bar: exposed).

Levels of C-reactive protein (CRP), haptoglobin (HPT) and fibrinogen were increased in the serum or plasma of UfCP exposed SHRs compared to control on 1^st^ recovery day (Figure 7; Table 2). CRP concentration increased by 58% (~1.6 fold, p < 0.05) in the serum (control: 132.7 ± 10.2 exposed: 209 ± 4.4 μg/ml); HPT concentration increased by 16% in the serum (control: 611 ± 17; exposed 710 ± 20 mg/dl, p < 0.05); and fibrinogen concentration increased by 13.6% (control: 191 ± 5; exposed 217 ± 8.0 mg/dl, p < 0.05) in plasma.

#### Effect on renin-angiotensin system (RAS)

Based on the cardiovascular response (altered BP and HR) in UfCP exposed aged SHRs plasma rennin and angiotensin (II) concentration and activity were assayed employing specific radioimmunoassay as described previously [32]. No effect on RAS was observed due to UfCP exposure in aged SHRs.

## Discussion

Inflammation, vascular reactivity, and atherosclerotic effects are the main contributing events for ambient ultrafines and UfCP exposure mediated onset of cardiovascular impairments [33-36]. In this study we have shown that UfCP inhalation exposure of aged SHRs results in an immediate increase in the levels of inflammatory cells and IL-6 in the BAL fluid and increased pulmonary expression for markers of inflammation, vasoconstriction and coagulation. The detected pulmonary response may be linked to the response observed on the systemic level, as evidenced by changes of cardiovascular parameters (BP, HR, HRV) and the increases in inflammatory mediators at the local and/or systemic level, the activation of blood coagulation mechanisms as evidenced by increased TF, fibrinogen, platelet count and acute phase reactant (CRP, HPT). Our findings further point towards the potential susceptibility characteristics and plausible molecular mechanisms which might increase the cardiovascular risk to aged SHRs following UfCP exposure.

### UfCP induced pulmonary and systemic effect on aged SHRs

SHRs have been widely used as an ideal animal model of hypertension to study the cardiovascular disease [37]. As the total peripheral resistance increases from normal to elevated levels, the SHRs progresses into the established hypertension state, typically, between the 4^th^ and 6^th^ months of age [38]. Hypertension is a commonly considered condition by epidemiologists in stratified analyses while studying the association between short-term PM exposure and cardiovascular-impairments. However, it is unclear whether preexisting hypertension modifies such associations. One study conducted in Utah found no evidence of increased risk for acute ischemic heart disease (IHD) events following PM2.5 exposure among individuals with preexisting hypertension compared with those without hypertension [39]. In contrast, Peel et al. [40] found that the presence of preexisting hypertension resulted in an increased risk of CVD with PM10 exposure. Similarly Rueckerl et al. [41] summarized the existing positive association between ambient air pollution and increased ischemic events in susceptible individuals. The potential effect of hypertension on the manifestation of ambient UfCP mediated cardiovascular effects is supported by a toxicological study conducted in a rat model of hypertension [42]. Another study demonstrated that concentrated ambient particles (CAPs) exposure resulted in higher mean arterial pressure compared to controls [43]. These experimental findings clearly indicate an association between PM2.5 exposure and hypertension leading to worsening of hypertension-related cardiovascular outcomes observed in epidemiological studies.

A series of epidemiological and in vivo studies have shown that exposure to UfCP is associated with pulmonary and/or systemic inflammation caused by deposition of particles in the alveoli or due to the translocation of fine particle into the systemic circulation [29,44-47] as the particle get smaller. Recently Kreyling et al. [17] have reported that translocation of UfCP across the air-blood barrier and accumulation in the secondary organs or tissues (heart, blood) are highly dependent on the particle specific surface area [17]. However Kreyling et al. [17,45] have also shown that particle mediated adverse health effects in secondary organs (cardiovascular system) are not only the result of particle translocation following exposure (inhalation/instillation); rather chemical composition, physical characteristics or particles (size, surface charges) are the important determinant to induce adverse health effect (cardiovascular impairments). Multiple mechanisms such as particle induced local and systemic inflammatory response, activation of oxidative stress, increased blood coagulation and endothelial dysfunction are likely to be involved in UfCP mediated cardiovascular events [48-50]. Alternatively, it is possible that increased sympathetic tone might also contribute to cardiophysiological response following an acute exposure to UfCP.

Increased BP and HR in association with HRV in aged SHRs are consistent with epidemiological and experimental observations. Significant decrease of SDNN on 1^st^ and 2^nd^ recovery day following 24 h exposure to UfCP represents overall depression of HRV, which reflects autonomic modulation of rhythmic heart rate. Similar response was reported by Devlin et al. [51] in a controlled experiment where significant decrease in HRV was observed in elderly individuals immediately after exposure to concentrated ambient particles. Consistent with our result, in another controlled study with predisposed (asthmatic) individuals Power et al. [52] reported significant decrease of SDNN immediately following exposure to particles and ozone. Measurement of HRV as 5 minutes SDNN decreased by 18-26% during the 10 minutes resting period in 11 healthy aged school crossing guards following exposure to heavy traffic area in their morning shift [53]. All these findings are highly comparable with our present result showing overall significant reduction of HRV in aged SHRs after exposure to UfCP. Additionally significant reduction of LF/HF ratio (29%) in aged SHRs is also supported by the finding from Timonen et al. [54]. The study involving pooled analysis of three centers (Erfurt, Amsterdam and Helsinki) demonstrated 13.5% reduction of LF/HF with a lag of 2 days following exposure to ambient PM_2.5_ [54]_._ Although, the physiological importance of observed changes in HRV is not fully known, reduced HRV has been associated with increased risk for developing coronary heart disease and to sudden cardiac death.

Increased level of BAL IL-6 in aged SHRs immediately following UfCP exposure may induce the risk of both venous and arterial thrombosis by increasing expression of tissue factor, fibrinogen and platelet activation [48]. The association between IL-6 and ischemic heart disease is well documented and is substantially attributable to the effects of IL-6 on atherosclerosis plaque formation. Mutlu et al. [48] thus provided a potential mechanism linking ambient PM exposure and thrombotic events. In that study authors have further shown that the induced pulmonary cytokine production (IL-6 from alveolar macrophages) following PM exposure might be responsible for increased intravascular thrombin formation, and accelerated arterial thrombosis. In a follow-up study Scott Budinger et al. [49] concluded that PM-induced lung inflammation or IL-6 generation is sufficient for the activation of coagulation and impaired fibrionolysis locally (lung) as well as systematically (blood, cardiac tissue etc.). IL-6 also induces the release of acute phase reactant (like CRP, HPT and fibrinogen) from hepatocytes [48,55]. HPT, fibrinogen, and CRP are the typical acute phase protein that are induced during inflammation and are used clinically as an indicator of the presence and intensity of inflammation. Increased IL-6, CRP, and fibrinogen levels are highly associated with increased risk of mortality from cardiovascular diseases as evidenced by Riediker et al. [56]. They also detected increased CRP levels in highway patrol officers after exposure to traffic-related PM. Similar correlation between PM exposure and serum CRP levels have been reported by Rueckerl and colleagues among predisposed men [57]. Furthermore Bourdon et al. [26] reported that inflammatory signaling molecules like IL-6 or CRP are involved in pulmonary injury and the trigger of the acute phase response (APR) following exposure to carbon black nano particles (CBNP). This in turn may further increase susceptibility to cardiovascular impairments.

A study by Ridker et al. [58] reported hs-CRP as the strongest univariate and only multivariate predictor for the risk of cardiovascular events in women among twelve markers measured. The median hs-CRP levels of women with cardiovascular events was 0.42 mg/dl whereas among women free of cardiovascular events the hs CRP levels were 0.28 mg/dl, i.e. 50% induction in diseased state. The fact that we observed a comparable increase in CRP levels (58%) in exposed aged SHRs supports the relevance of our findings for cardiovascular events. Most of the animal studies also provide strong evidence of increased CRP levels following PM exposure [59,60]. Significantly stronger PM-induced CRP responses were observed in SHRs, compared to rats with normal blood pressure [61]. Recently Higashisaka et al. [60] have shown a significant increase of CRP and HPT in BALB/c mice treated with silica nanoparticles. Consistent to the findings of other researchers [60,61], we also found significant induction of CRP, fibrinogen and HPT in aged SHRs following inhalation exposure to UfCP. We have previously shown that CRP and HPT were not affected in adult SHRs after exposure to UfCP (Table 2; [31]). Recently Farina et al. [62] reported that the translocation of inflammatory mediators, such as cytokines and UfCP from lungs to bloodstream might trigger a systemic reaction resulting in endothelial activation, oxidative stress and subsequent cardiovascular response. The significant induction of systemic acute phase reactant (CRP, HPT) and coagulation markers (fibrinogen and platelet) might be associated with UfCP mediated release of IL-6 from alveolar macrophages as well as translocation of UfCP from lung to the systemic circulation as previously suggested by several researchers [46,48,49,62]. Following 2 h controlled exposure of 19 predisposed subjects (type II diabetes) to elemental carbon UfCP, Stewart et al. [35] reported transient activation of blood platelets, with possible activation of blood leukocytes and vascular endothelium. From this study authors have further inferred that the observed transient and small effect can even lead to acute vascular insult with prothrombotic consequences that could increase the risk and susceptibility for immediate UfCP induced cardiovascular events among elderly individuals with preexisting complications like hypertension, diabetes [5,6].

Induction of ET(A) along with induced pulmonary expression of ET-1 in aged SHRs after UfCP exposure may cause pulmonary microvascular endothelial dysfunction. Holcox et al. [63] suggested endogenous endothelin by stimulation of ET(A) receptor contribute to basal constrictor tone and endothelial dysfunction. Chauhan et al. [64] have shown up-regulation of ET-1 in human pulmonary epithelial cells (A549) following exposure to the ambient PM. Our findings of ET-1 up-regulation in UfCP exposed aged SHRs are in agreement with Karthikeyan et al. [65], who observed induction of ET-1 in association with pulmonary inflammation and oxidative stress in rats after exposure to diesel exhaust particle. Interestingly, reduced ET(B) expression have been observed in UfCP exposed aged SHR lungs. Kobayshi et al. [66] suggested that down-regulation of ET(B) receptor, but not ET(A) receptor, occurs in the congestive rat lung. Kobayshi et al. [66] further proposed that down-regulation of lung ET(B) receptors partially contributes to the significant increase in ET-1 and may be involved in the progression of pulmonary injury in aged SHRs. Indeed, increased level ET-1 in UfCP exposed pulmonary tissue may increase oxidative stress, pulmonary neutrophils activation after UfCP exposure. In the heart, ET-1 is synthesized by cardiomyocytes, fibroblasts, and endothelial cells and it directly stimulates cardiac fibroblasts to produce extracellular matrix proteins thus promoting myocardial fibrosis [67]. Hence, the increased expression of ET-1 in cardiac tissue of aged SHRs might be responsible for alteration of cardiac physiological measures.

In the cardiac tissue of aged SHRs, over expression of only ET-1 might be an indirect effect of systemic inflammatory changes. However, Bourdon et al. [68] recently suggested that CBNP or UfCP mediated systemic inflammatory effects are likely responsible events for cardiovascular events without any direct alteration of gene expression in the heart tissue. They showed that gene expression in the heart remained unaffected by CBNP exposure in spite of substantial alteration of pulmonary and hepatic gene expression along with increased plasma cell adhesion molecules and PA-I. However the distinct differences between present study and Bourdon et al. [68] study should be accounted. As for example, different species (healthy mice versus compromised rats) and UfCP instillation of aggregated UfCP at a relative high dosage of 162 μg for mice versus inhalation over 24 h in our study with a cumulative estimated dose of 31 μg in rats (tidal volume 2 ml, breathing frequency 120 min^−1^, respiration 0.345 m^3^/day, 180 μg/m^3^ UfCP, deposition efficiency 50% [69]).

Previously we have shown an induction of HO-1 in adult SHRs with a lag of 3 days following 24 h exposure to UfCP [31]. In this study involving aged SHRs, the significant induction of lung HO-1 occurred immediately after exposure to UfCP suggests an earlier onset of oxidative stress. Overproduction of reactive oxygen species (ROS) under pathophysiologic conditions forms an integral part of the development of cardiopulmonary disease, and in particular atherosclerosis plaque formation [70,71]. Increased expression of HO-1 is an indication of oxidative stress, and therefore assessment of HO-1 levels may assist in identifying the susceptible individuals for UfCP exposure related adverse health effects [72]. The induction of HO-1 in the lung might suggest an induction of oxidative stress from UfCP exposure [73].

In this study we detected inflammatory effects, cardiovascular and acute phase responses after exposure to a rather low mass dose of 31 μg UfCP deposited in the rat lungs throughout the 24 h exposure (see calculation above). Based on these estimates biomarkers of inflammation and acute phase response are increased at a considerable low mass dosage. However, for UFP the large particle surface area available for interaction with biological materials rather than the particle mass has turned out to be the key determinant of toxicity [74]. Accordingly, an approximation of the UfCP surface area deposited in the rat lung reveals 0.024 m^2^ throughout the 24 h exposure (respiration of 0.345 m^3^/day, 0.139 m^2^ UfCP surface/m^3^, deposition efficiency 50% [69]). If a human would inhale the applied UfCP at the same concentration for one day, the lung burden would be 1.46 m^2^ in terms of UfCP surface area or 1.89 mg in terms of mass (15 m^3^/day inhaled air, deposition efficiency 70%, [75]. However, a rough estimate for urban sites of high air pollution close to major traffic roads suggests the daily lung burden for urban residents to be about 0.015 m^2^/day or 0.14 mg, assuming that UFPs in urban air are derived mainly from mobile sources with a specific surface area comparable to DEP (110 m^2^/g) and a UFP mass concentration of 13 μg/m^3^ [76]. Thus, the UfCP surface area dose delivered experimentally to the rat lung is comparable to that urban residents may undergo. However, the alveolar surface area differs by a factor of ~100 between the two species. Thus the experimentally delivered particle surface area clearly exceeds that estimated for urban residents. Considering the deposited UFP which may accumulate in the lung over time [77,78], the experimentally delivered surface dose under environmental conditions could be reached within months thereby supporting the notion that biological effects in our experimental setting are detectable at a relative low dosage.

### Comparison of susceptibility between aged and adult SHRs

The term susceptible or sensitive population has been used more generally to characterize the response of human to air pollutants [6]. Collective evidence from several epidemiological studies as well as control human exposure and animal studies have identified certain characteristics which may lead to increased susceptibility to air pollutant or UfCP mediated health risk [1,11]. They include life stages, genetic polymorphisms, preexisting conditions like hypertension, respiratory diseases, and diabetic condition [5,6,35,79,80]. However, supporting experimental evidence is required to identify the mechanisms by which each of these factors change the susceptibility and leading to a higher degree of risk to PM-related health effects in one population compared to another.

We have previously shown that 24 h inhalation exposure of adult SHRs to UfCP significantly increases HR and BP with a lag 1–3 days without PMN influx [31]. In contrast, under similar exposure conditions, transient increases of HR with moderate pulmonary neutrophilic influx were detected in healthy WKY rats [81]. In the present study, we detected small but significant pulmonary inflammation along with release of systemic acute phase reactants (CRP, HPT) and induced coagulatory pathways (fibrinogen, tissue factor) in aged SHRs following UfCP exposure. Previous studies have shown that young adult SHR are less sensitive to neutrophilic inflammation compared to WKY when exposed to combustion source particles by inhalation [82,83]. It is possible that at an older age, SHR respond more readily to UfCP than at younger age with regards to the neutrophilic inflammation.

Our earlier study involving adult SHRs showed increases in BP and HR with marked increases in lung expression of vasoconstriction and clotting markers with a lag of 1–3 days after UfCP in the absence of significant pulmonary neutrophil influx [31]. The early induction of these changes in aged SHR following 24 h exposure to UfCP together with significant pulmonary neutrophil influx and also systemic increases in thrombosis biomarkers indicates that aged SHRs may experience greater cardiovascular morbidity from exposure to UfCP when compared to adult SHR and that there is likely a shift in the time-lag, important to consider in epidemiological studies. The biological significance of the dissociation in the time course between aged and adult SHRs cannot be directly inferred from our exposure scenario. However, one may speculate that the diminished compensatory antioxidant reserve in aged SHR may predispose them to early expression of changes while in the adult rats UfCP-induced changes might be countered for a longer duration. A detailed comparison of all observed parameters (cardiovascular, inflammatory) from aged and adult SHRs following exposure to UfCP has been provided in Table 2, which clearly indicates that aged individuals are showing more UfCP induced health impact and a different response pattern than the predisposed adult individuals or young healthy WKY rats [81]. In line, epidemiological studies in some cities of Australia and New Zealand by Barnett et al. [80] showed a significant association between increased air pollution and higher cardiovascular impairment related hospital admission of elderly individuals with IHD and MI which were not apparent for younger individuals. Comparison of cardiovascular response patterns in aged compromised animals (i.e. SHRs) with healthy WKY rats [81] and adult SHRs [31] suggests that aged hypertensive animals are more susceptible to UfCP that might have been contributed due to their worsening cardiovascular complications at an advanced age.

Compared to our previous study [31], rennin-angiotensin concentration remained unaffected in aged SHRs following exposure to UfCP. Aged SHR may differ from that of adult SHR for their regulation of renin-angiotensin-aldosterone system associated with advanced kidney damage although renin-angiotensin concentrations remain unaffected. UfCP exposure was associated with induction of TF (1.5-fold) in the lungs of aged SHRs, which might cause activation of coagulation cascade promoting thrombus formation and the risk of cardiac events [50,84]. In adult SHRs, we previously reported induction of lung TF following 72 h of UfCP exposure. Budinger and colleagues [49] observed an IL-6 dependent increase in lung TF transcripts following exposure to concentrated ambient particles. Comparison of our present findings (aged SHRs) with previous observation (adult SHRs) are consistent with a large body of epidemiological and clinical/toxicological studies suggesting that elderly individuals with preexisting pathophysiological conditions are likely more vulnerable to PM mediated health effects [6,85].

## Conclusion

Exposure of aged SHRs to UfCP induced a neutrophilic influx and an increased IL6 level in the lungs. This inflammatory response of the lungs is paralleled by a systemic inflammation indicated by increased CRP, HPT, fibrinogen levels, and oxidative stress as inferred from increased HO-1 expression in the lung. Further, increased pulmonary expression of endothelin and systemic markers of blood coagulation are suggestive for a disturbed vasoregulation and coagulatory homeostasis. Contemporaneous cardiovascular impairments (induced BP and HR) in the aged animals suggest the contribution of vasoconstrictive and thrombogenic effects in observed physiological impairment in the heart. The response we observed in aged SHR differs from that of adult SHRs, in that UfCP exposures mediated an increase in BP and HR occurred without a traceable pulmonary and systemic inflammation [31]. This suggests that aged SHRs are likely to be more susceptible than the adult SHRs to UfCP mediated cardiovascular risks and that there is likely a differential time lag for adult and aged rats. Based on our data, we cannot disentangle the contribution of age versus advanced disease in the observed systemic and cardiovascular effects. Nevertheless, the complex response observed in the lungs and on a systemic level in the aged, cardiovascular compromised animals provides further evidence for potential mechanisms explaining the increased risk of particle mediated cardiac health effects in the cardiovascular compromised elderly.

## Materials and methods

### Animals

Aged male spontaneously hypertensive rats (SHR; 12 months, body weight: 411 ± 4.7 g; n = 15) were used for the present study. Animals were housed under filtered air and specific pathogen free (SPF) conditions at a mean temperature of 22 ± 2°C, a mean relative humidity of 50 ± 5%, and a 12 h light–dark cycle (6 a.m. to 6 p.m. light on) with pelleted feed and filtered water being supplied *ad libitum*. Experimental protocols were approved by the Animal Care and Use Committee of the Helmholtz Zentrum München – German Research Center for Environmental Health and by the Bavarian Animal Research Authority (211-2531-88/2001).

### Ultrafine carbon particle generation and whole body exposure chamber

The methodology of UfCP generation and the setup of the whole body exposure system for rodent have been previously described [31,81]. UfCP showed a monomodal number distribution in the exposure chamber with a median particle size ± arithmetic SD of 31 ± 0.3 nm. Measured mass and number concentration was 180 μg m^−3^ and 9× 10^6^ cm^−3^, respectively. This translates into a surface area concentration of 0.139 m^2^ (particle) m^−3^ (air) because the mass specific surface area (according to the BET method) of the UfCP was determined to be 807 m^2^ g^-1^. Based on the polydispersity of the particle distribution (geometric standard deviation is 1.51) a median mass diameter of 46 nm is calculated. An approximation of the dose deposited in the rat lung reveals 31 μg UfCP or 0.024 m^2^ UfCP surface areas throughout the 24 h exposure. This dose was selected with respect to peak ambient UFP concentrations which may accumulate a corresponding dose in the lungs of healthy humans in a reasonable time, i.e. within several months (see Discussion).

### Experimental design

The cardiophysiological responses, i.e. effects on HR and BP were measured on aged SHRs (n = 7) following 24 h UfCP inhalation exposure using a radiotelemetry system. Although BP and HR were increased only on 1^st^ day of recovery, however, subsequent exposures were conducted in additional, non-telemetry SHRs to obtain blood, BAL and tissue samples on both 1^st^ and 3^rd^ day of recovery following 24 h of exposure as in our previous study [31].

Each exposure used 16 SHRs, 8 animals were exposed to filtered air (controls) while the other 8 animals were exposed to UfCP for 24 h (exposed). In the first study, animals were sacrificed in the morning of the 1^st^ day of recovery, similarly in a separate study another 16 animals were sacrificed 72 h or on 3^rd^ day of recovery following 24 h exposure to UfCP. Prior to BAL collection, blood samples of 8 SHRs were collected from retro orbital sinus for analysis of haematological parameters and from the abdominal aorta for analysis of biomarkers. Six animals were used to collect BAL and tissue samples (heart and lung) for further assessment of pulmonary and systemic response, the remaining 2 SHRs of each group were used for pulmonary and cardiac histopathology. Animal distribution and sample collection of the control group was similar to that of the exposed group.

### Cardiophysiological analysis by radiotelemetry

#### Animal preparation (Surgical implantation)

The implantation of telemetric devices into the peritoneal cavity of animals was performed as previously described [31,81]. After 10 days of post surgical recovery, two days (day -2 and day -1) acclimatization of animals in the exposure chamber had been carried out prior to the actual data recording cardiovascular response (BP and HR) reaches to its baseline values. Data recording was then initiated and continued for six days (Figure 1), that included a baseline reading (day 0), exposure (day 1), and recovery period readings (days 2–5).

### Animal exposure

Individual animals served as their own controls similar to our previous study [31]. Following 10 days of post surgical recovery and 2 days of acclimatization in the exposure chamber aged SHRs (n = 7) were primarily exposed to filtered air (control) and 4 weeks later to UfCP by whole-body exposure for 24 hrs. A time gap of 4 weeks was chosen in between control (filtered air) and UfCP exposure to ensure elimination of any possible effects of clean air exposure as individual animals served as their own control. However, comparison of baseline values between 12 and 13 months old SHRs indicate that cardiac performance was not altered by the 4 week time gap between control and exposure conditions (see baseline values in results).

### Data acquisition

Cardiophysiological response before and after inhalation exposure to UfCP was performed on 12 months old SHRs (n = 7) by using radio telemetric system as described in our previous study [31,81]. Briefly arterial BP, HR, T, and Act of SHRs were continuously collected over 24 h/day, throughout baseline, exposure and recovery periods. Arterial blood pressure (sBP, dBP, and mBP) were determined from the BP tracings on a beat to beat basis. For HRV analysis, each 5-minutes ECG segment per hour was randomly selected for 12 h dark period. Standard deviation of all adjacent normal sinus NN intervals (SDNN) for each of those 5-minutes segments were considered as overall HRV. Additionally the square root of the mean of squared differences between adjacent normal to normal intervals (RMSSD) and the low-frequency to high-frequency ratio (LF/HF) was determined, which reflects the balance of cardiac parasympathetic tone and sympathetic activity, respectively. The detailed methodology of HRV data analysis is as described previously (81).

### Assessment of UfCP mediated pulmonary inflammatory response

#### BAL and lung tissue processing

BAL analysis was performed as described in our previous studies [31,81]. One aliquot of whole BAL (n = 6) was used for determining total cell counts (Coulter Counter; Coulter, Inc., Miami, FL), and a second aliquot was centrifuged (Cytospin 2; Shandon, Astmoor, UK) to counts cell differential. Macrophages, polymorphonuclear cells (PMNs, or neutrophil), eosinophil, and lymphocyte were counted using light microscopy (over 200 cells counted per slide). The remaining BAL was centrifuged (1500 × g) to remove cells, and the supernatant fluids were used to analyze the protein, albumin, γ-Glutamyltransferase (GGT), N-acetyl glucosaminidase (NAG) activity, IL-6 as potential biological markers for pulmonary inflammation, capillary leakage and lung injury. Furthermore, lung and left ventricular tissues were snap frozen in liquid nitrogen for later RNA extraction and RT-PCR for markers associated with pulmonary inflammation, oxidative stress, vasoconstriction and thrombosis (n = 6).

### Pulmonary histopathology

Pulmonary histopathology was performed as previously described [31,81].

### Assessment of UfCP mediated effects on pulmonary and cardiac tissue

#### Gene expression

Lung and cardiac total RNA was isolated using the RNeasy (lung) or RNeasy fibrous tissue (heart) kits and protocols from Qiagen. RNasin Plus (Promega), a broad spectrum RNase inhibitor, was added to each sample immediately after isolation from the tissue. Concentration and purity of the RNA samples were determined with the NanoDrop ND-100 spectrophotometer (NanoDrop Technologies). Aliquots of each sample were diluted to a concentration suitable for PCR stored at −80°C. Relative quantification of gene expression was determined by real-time RT-PCR using the Applied Biosystems Inc. model ABI 7900 HT Sequence Detection System, as described previously [31]. Gene specific primers for control and target genes were purchased from Applied Biosystems Incorporated [31]. SuperScript III Platinum One-Step Quantitative RT-PCR System from Invitrogen reagent kit was used for expression assays. Reverse transcription and amplification conditions were as follows: 53°C for 20-minutes, 95°C for 2-minutes, and 40 cycles at 95°C for 15 seconds and 60°C for 45 seconds. Data were analyzed using ABI sequence detection software (SDS), version 2.2. For each PCR plate, cycle threshold (Ct) was set to an order of magnitude above background. For each individual sample, target gene Ct was normalized to a control Ct to account for variability in starting RNA amount. Expression of each exposure group was quantified as fold difference over FA control, at the corresponding time point.

#### Cardiac histopathology

Cardiac histopathology was performed as previously described [31,81].

### Assessment of UfCP mediated systemic response

To assess the UfCP mediated inflammatory response at systemic level blood samples of each animal were collected from retro orbital sinus (haematology) and from abdominal aorta (biomarkers) on 1^st^ and 3^rd^ day of recovery.

#### Haematology

500 μl of blood sample from retro orbital sinus of each animal (n = 8) was collected in EDTA-Microvette for hematological analysisusing haematology analyzer (Bayer ADVIA 120, Germany).

#### Acute phase protein analysis

Blood samples collected from abdominal aorta (blood A, n = 8) were separated citrate. Each citrated blood sample was then centrifuged (at 2710 g) for 10-minutes (4°C) and plasma samples were stored at −80°C until analyzed. Fibrinogen concentration was measured in each citrated plasma samples as previously described [86]. Serum was collected from blood samples by centrifugation for 15-minutes (at 1300 g, 4°C). Serum CRP was measured using the kit from DiaSorin Inc. (Stillwater, MN) and HPT using the kit from Kamiya Biomedical Company (Seattle, WA).

Renin/Angiotensin analysis: Renin, angiotensin I (Ang I) and angiotensin II (Ang II) concentrations and activity were measured from plasma following 24 h of exposure to UfCP. For this purpose blood samples were collected from abdominal aorta (n = 8) in a 5 ml falcon tube containing mixture (140 μl/ml blood) of p-hydroxy-mercuribenzoic acid (10 μl, Sigma), phenyl-methyl-sulfonyl-fluoride (10 μl, Sigma), EDTA (50 μl), pepstatin A (20 μl, Sigma) and o-phenanathrolin (50 μl, Merck). Measurements of renin activity and concentration as well as angiotensin (I and II) concentrations were assayed employing specific radioimmunoassay, as previously described [32].

### Statistics

After checking for the normal distribution assumption the differences between exposure and control groups were compared by using the *t*-test. Cardiovascular response parameters were described by a linear mixed regression model for repeated measurements. Based on this model group differences between the exposure and control group were tested. For expression analysis of various parameters from lung and heart tissues, a two-way analysis of variance (ANOVA) was used to analyze differences between the groups. However, for the plasma rennin and angiotensin (I and II) data the normality assumption did not hold. Therefore, the Wilcoxon rank sum test was performed for plasma rennin and angiotensin (I and II) concentration. P values less than 0.05 were considered as statistically significant. All computations were done by the software packages Statgraphics plus v 5.0 (Manugistics, Rockville, MD) and SAS V9.1 (Cary, NC). Data are presented as arithmetic mean values of n observations ± the standard error (SE), unless otherwise indicated.

## References

[1] Hoek G, Krishnan RM, Beelen R, Peters A, Ostro B, Brunekreef B, Kaufman JD (2013). Long-term air pollution exposure and cardio- respiratory mortality: a review. Environ Health.

[2] Prüss-Üstün A, Corvalán C: ***Preventing disease through healthy environments.*** *Towards an estimate of the environmental burden of disease.* Geneva: WHO; 2006.

[3] Rich DQ, Kipen HM, Huang W, Wang G, Wang Y, Zhu P, Ohman-Strickland P, Hu M, Philipp C, Diehl SR, Lu SE, Tong J, Gong J, Thomas D, Zhu T, Zhang JJ (2012). Association between changes in air pollution levels during the Beijing Olympics and biomarkers of inflammation and thrombosis in healthy young adults. JAMA.

[4] Brook RD, Franklin B, Cascio W, Hong Y, Howard G, Lipsett M, Luepker R, Mittleman M, Samet J, Smith SC, Tager I (2004). Air pollution and cardiovascular disease: a statement for healthcare professionals from the Expert Panel on Population and Prevention Science of the American Heart Association. Circulation.

[5] Nichols JL, Owens EO, Dutton SJ, Luben TJ (2013). Systematic review of the effects of black carbon on cardiovascular disease among individuals with pre-existing disease. Int J Public Health.

[6] Sacks JD, Stanek LW, Luben TJ, Johns DO, Buckley BJ, Brown JS, Ross M (2011). Particulate matter-induced health effects: who is susceptible?. Environ Health Perspect.

[7] AQMP (2012). Air quality management plan revised draft.

[8] **Healthy eating index-2010 reports.** [http://www.cnpp.usda.gov/HealthyEatingIndex.htm]

[9] Barraza-Villarreal A, Escamilla-Nunez MC, Hernandez-Cadena L, Texcalac-Sangrador JL, Sienra-Monge JJ, Del Rio-Navarro BE, Cortez-Lugo M, Sly PD, Romieu I (2011). Elemental carbon exposure and lung function in school children from Mexico City. Eur Respir J.

[10] Barraza-Villarreal A, Sunyer J, Hernandez-Cadena L, Escamilla-Nunez MC, Sienra-Monge JJ, Ramirez-Aguilar M, Cortez-Lugo M, Holguin F, Diaz-Sanchez D, Olin AC, Romieu I (2008). Air pollution, airway inflammation, and lung function in a cohort study of Mexico City schoolchildren. Environ Health Perspect.

[11] Nemmar A, Holme JA, Rosas I, Schwarze PE, Alfaro-Moreno E (2013). Recent advances in particulate matter and nanoparticle toxicology: a review of the in vivo and in vitro studies. Biomed Res Int.

[12] Khandoga A, Stoeger T, Khandoga AG, Bihari P, Karg E, Ettehadieh D, Lakatos S, Fent J, Schulz H, Krombach F (2010). Platelet adhesion and fibrinogen deposition in murine microvessels upon inhalation of nanosized carbon particles. J Thromb Haemost.

[13] Schwarze PE, Totlandsdal AI, Lag M, Refsnes M, Holme JA, Ovrevik J (2013). Inflammation-related effects of diesel engine exhaust particles: studies on lung cells in vitro. Biomed Res Int.

[14] Chalupa DC, Morrow PE, Oberdorster G, Utell MJ, Frampton MW (2004). Ultrafine particle deposition in subjects with asthma. Environ Health Perspect.

[15] Daigle CC, Chalupa DC, Gibb FR, Morrow PE, Oberdorster G, Utell MJ, Frampton MW (2003). Ultrafine particle deposition in humans during rest and exercise. Inhal Toxicol.

[16] Wallenborn JG, McGee JK, Schladweiler MC, Ledbetter AD, Kodavanti UP (2007). Systemic translocation of particulate matter-associated metals following a single intratracheal instillation in rats. Toxicol Sci.

[17] Kreyling WG, Hirn S, Moller W, Schleh C, Wenk A, Celik G, Lipka J, Schaffler M, Haberl N, Johnston BD, Sperling R, Schmid G, Simon U, Parak WJ, Semmler-Behnke M (2014). Air-blood barrier translocation of tracheally instilled gold nanoparticles inversely depends on particle size. ACS Nano.

[18] Kreyling WG, Hirn S, Schleh C (2010). Nanoparticles in the lung. Nat Biotechnol.

[19] Stoeger T, Schmid O, Takenaka S, Schulz H (2007). Inflammatory response to TiO2 and carbonaceous particles scales best with BET surface area. Environ Health Perspect.

[20] Sager TM, Kommineni C, Castranova V (2008). Pulmonary response to intratracheal instillation of ultrafine versus fine titanium dioxide: role of particle surface area. Part Fibre Toxicol.

[21] Geiser M, Rothen-Rutishauser B, Kapp N, Schurch S, Kreyling W, Schulz H, Semmler M, Im Hof V, Heyder J, Gehr P (2005). Ultrafine particles cross cellular membranes by nonphagocytic mechanisms in lungs and in cultured cells. Environ Health Perspect.

[22] Frampton MW, Stewart JC, Oberdorster G, Morrow PE, Chalupa D, Pietropaoli AP, Frasier LM, Speers DM, Cox C, Huang LS, Utell MJ (2006). Inhalation of ultrafine particles alters blood leukocyte expression of adhesion molecules in humans. Environ Health Perspect.

[23] BéruBé K, Balharry D, Sexton K, Koshy L, Jones T (2007). Combustionderived nanoparticles: mechanisms of pulmonary toxicity. Clin Exp Pharmacol Physiol.

[24] Stoeger T, Takenaka S, Frankenberger B, Ritter B, Karg E, Maier K, Schulz H, Schmid O (2009). Deducing in vivo toxicity of combustionderived nanoparticles from a cell-free oxidative potency assay and metabolic activation of organic compounds. Environ Health Perspect.

[25] Su DS, Jentoft RE, Muller JO, Rothe D, Jacob E, Simpson CD, Tomovic Z, Mullen K, Messerer A, Poschl U, Niessner R, Schlogl R (2004). Microstructure and oxidation behaviour of Euro IV diesel engine soot: a comparative study with synthetic model soot substances. Catal Today.

[26] Bourdon JA, Halappanavar S, Saber AT, Jacobsen NR, Williams A, Wallin H, Vogel U, Yauk CL (2012). Hepatic and pulmonary toxicogenomic profiles in mice intratracheally instilled with carbon black nanoparticlesreveal pulmonary inflammation, acute phase response, and alterations in lipid homeostasis. Toxicol Sci.

[27] Jacobsen NR, Moller P, Jensen KA, Vogel U, Ladefoged O, Loft S, Wallin H (2009). Lung inflammation and genotoxicity following pulmonary exposure to nanoparticles in ApoE−/− mice. Part Fibre Toxicol.

[28] Karg E, Roth C, Heyder J (1998). Do inhaled ultrafine particles cause acute health effects in rats? II: Exposure system. J Aerosol Sci.

[29] Brook RD, Rajagopalan S, Pope CA, Brook JR, Bhatnagar A, Diez-Roux AV, Holguin F, Hong Y, Luepker RV, Mittleman MA, Peters A, Siscovick D, Smith SC, Whitsel L, Kaufman JD (2010). Particulate matter air pollution and cardiovascular disease: an update to the scientific statement from the American Heart Association. Circulation.

[30] Gold DR, Mittleman MA (2013). New insights into pollution and the cardiovascular system: 2010 to 2012. Circulation.

[31] Upadhyay S, Stoeger T, Harder V, Thomas RF, Schladweiler MC, Semmler-Behnke M, Takenaka S, Karg E, Reitmeir P, Bader M, Stampfl A, Kodavanti UP, Schulz H (2008). Exposure to ultrafine carbon particles at levels below detectable pulmonary inflammation affects cardiovascular performance in spontaneously hypertensive rats. Part Fibre Toxicol.

[32] Hermann K, Ganten D, Unger T, Bayer C, Lang RE (1988). Measurement and characterization of angiotensin peptides in plasma. Clin Chem.

[33] Miller MR, Shaw CA, Langrish JP (2012). From particles to patients: oxidative stress and the cardiovascular effects of air pollution. Future Cardiol.

[34] Sager TM, Wolfarth MW, Battelli LA, Leonard SS, Andrew M, Steinbach T, Endo M, Tsuruoka S, Porter DW, Castranova V (2013). Investigation of the pulmonary bioactivity of double-walled carbon nanotubes. J Toxicol Environ Health A.

[35] Stewart JC, Chalupa DC, Devlin RB, Frasier LM, Huang LS, Little EL, Lee SM, Phipps RP, Pietropaoli AP, Taubman MB, Utell MJ, Frampton MW (2010). Vascular effects of ultrafine particles in persons with type 2 diabetes. Environ Health Perspect.

[36] Chang CC, Hwang JS, Chan CC, Cheng TJ (2007). Interaction effects of ultrafine carbon black with iron and nickel on heart rate variability in spontaneously hypertensive rats. Environ Health Perspect.

[37] Pinto YM, Paul M, Ganten D (1998). Lessons from rat models of hypertension: from Goldblatt to genetic engineering. Cardiovasc Res.

[38] Doggrell SA, Brown L (1998). Rat models of hypertension, cardiac hypertrophy and failure. Cardiovasc Res.

[39] Pope CA, Muhlestein JB, May HT, Renlund DG, Anderson JL, Horne BD (2006). Ischemic heart disease events triggered by short-term exposure to fine particulate air pollution. Circulation.

[40] Peel JL, Metzger KB, Klein M, Flanders WD, Mulholland JA, Tolbert PE (2007). Ambient air pollution and cardiovascular emergency department visits in potentially sensitive groups. Am J Epidemiol.

[41] Ruckerl R, Schneider A, Breitner S, Cyrys J, Peters A (2011). Health effects of particulate air pollution: a review of epidemiological evidence. Inhal Toxicol.

[42] Upadhyay S, Ganguly K, Stoeger T, Semmler-Bhenke M, Takenaka S, Kreyling WG, Pitz M, Reitmeir P, Peters A, Eickelberg O, Wichmann HE, Schulz H (2010). Cardiovascular and inflammatory effects of intratracheally instilled ambient dust from Augsburg, Germany, in spontaneously hypertensive rats (SHRs). Part Fibre Toxicol.

[43] Sun Q, Yue P, Ying Z, Cardounel AJ, Brook RD, Devlin R, Hwang JS, Zweier JL, Chen LC, Rajagopalan S (2008). Air pollution exposure potentiates hypertension through reactive oxygen species-mediated activation of Rho/ROCK. Arterioscler Thromb Vasc Biol.

[44] Terzano C, Di Stefano F, Conti V, Graziani E, Petroianni A (2010). Air pollution ultrafine particles: toxicity beyond the lung. Eur Rev Med Pharmacol Sci.

[45] Kreyling WG, Semmler-Behnke M, Seitz J, Scymczak W, Wenk A, Mayer P, Takenaka S, Oberdorster G (2009). Size dependence of the translocation of inhaled iridium and carbon nanoparticle aggregates from the lung of rats to the blood and secondary target organs. Inhal Toxicol.

[46] Kodavanti UP, Schladweiler MC, Gilmour PS, Wallenborn JG, Mandavilli BS, Ledbetter AD, Christiani DC, Runge MS, Karoly ED, Costa DL, Peddada S, Jaskot R, Richards JH, Thomas R, Madamanchi NR, Nyska A (2008). The role of particulate matter-associated zinc in cardiac injury in rats. Environ Health Perspect.

[47] Gilmour PS, Nyska A, Schladweiler MC, McGee JK, Wallenborn JG, Richards JH, Kodavanti UP (2006). Cardiovascular and blood coagulative effects of pulmonary zinc exposure. Toxicol Appl Pharmacol.

[48] Mutlu GM, Green D, Bellmeyer A, Baker CM, Burgess Z, Rajamannan N, Christman JW, Foiles N, Kamp DW, Ghio AJ, Chandel NS, Dean DA, Sznajder JI, Budinger GR (2007). Ambient particulate matter accelerates coagulation via an IL-6-dependent pathway. J Clin Invest.

[49] Budinger GR, McKell JL, Urich D, Foiles N, Weiss I, Chiarella SE, Gonzalez A, Soberanes S, Ghio AJ, Nigdelioglu R, Mutlu EA, Radigan KA, Green D, Kwaan HC, Mutlu GM (2011). Particulate matter-induced lung inflammation increases systemic levels of PAI-1 and activates coagulation through distinct mechanisms. PLoS One.

[50] Steffel J, Luscher TF, Tanner FC (2006). Tissue factor in cardiovascular diseases: molecular mechanisms and clinical implications. Circulation.

[51] Devlin RB, Ghio AJ, Kehrl H, Sanders G, Cascio W (2003). Elderly humans exposed to concentrated air pollution particles have decreased heart rate variability. Eur Respir J Suppl.

[52] Power KL, Balmes J, Solomon C (2008). Controlled exposure to combined particles and ozone decreases heart rate variability. J Occup Environ Med.

[53] Fan ZT, Meng Q, Weisel C, Laumbach R, Ohman-Strickland P, Shalat S, Hernandez MZ, Black K (2009). Acute exposure to elevated PM2.5 generated by traffic and cardiopulmonary health effects in healthy olderadults. J Expo Sci Environ Epidemiol.

[54] Timonen KL, Vanninen E, de Hartog J, Ibald-Mulli A, Brunekreef B, Gold DR, Heinrich J, Hoek G, Lanki T, Peters A, Tarkiainen T, Tiittanen P, Kreyling W, Pekkanen J (2006). Effects of ultrafine and fine particulate and gaseous air pollution on cardiac autonomic control in subjects with coronary artery disease: the ULTRA study. J Expo Sci Environ Epidemiol.

[55] Gauldie J, Northemann W, Fey GH (1990). IL-6 functions as an exocrine hormone in inflammation. Hepatocytes undergoing acute phase responses require exogenous IL-6. J Immunol.

[56] Riediker M, Cascio WE, Griggs TR, Herbst MC, Bromberg PA, Neas L, Williams RW, Devlin RB (2004). Particulate matter exposure in cars is associated with cardiovascular effects in healthy young men. Am J Respir Crit Care Med.

[57] Ruckerl R, Ibald-Mulli A, Koenig W, Schneider A, Woelke G, Cyrys J, Heinrich J, Marder V, Frampton M, Wichmann HE, Peters A (2006). Air pollution and markers of inflammation and coagulation in patients with coronary heart disease. Am J Respir Crit Care Med.

[58] Ridker PM, Hennekens CH, Buring JE, Rifai N (2000). **C-reactive protein and other markers of inflammation inthe prediction of cardiovascular** disease in women. N Engl J Med.

[59] Saber AT, Lamson JS, Jacobsen NR, Ravn-Haren G, Hougaard KS, Nyendi AN, Wahlberg P, Madsen AM, Jackson P, Wallin H, Vogel U (2013). Particle-induced pulmonary acute phase response correlates with neutrophil influx linking inhaled particles and cardiovascular risk. PLoS One.

[60] Higashisaka K, Yoshioka Y, Yamashita K, Morishita Y, Fujimura M, Nabeshi H, Nagano K, Abe Y, Kamada H, Tsunoda S, Yoshikawa T, Itoh N, Tsutsumi Y (2011). Acute phase proteins as biomarkers for predicting the exposure and toxicity of nanomaterials. Biomaterials.

[61] Rohr AC, Wagner JG, Morishita M, Kamal A, Keeler GJ, Harkema JR (2010). Cardiopulmonary responses in spontaneously hypertensive and Wistar-Kyoto rats exposed to concentrated ambient particles from Detroit, Michigan. Inhal Toxicol.

[62] Farina F, Sancini G, Battaglia C, Tinaglia V, Mantecca P, Camatini M, Palestini P (2013). Milano summer particulate matter (PM10) triggers lung inflammation and extra pulmonary adverse events in mice. PLoS One.

[63] Halcox JP, Nour KR, Zalos G, Quyyumi AA (2007). Endogenous endothelin in human coronary vascular function: differential contribution of endothelin receptor types A and B. Hypertension.

[64] Chauhan V, Breznan D, Thomson E, Karthikeyan S, Vincent R (2005). Effects of ambient air particles on the endothelin system in human pulmonary epithelial cells (A549). Cell Biol Toxicol.

[65] Karthikeyan S, Thomson EM, Kumarathasan P, Guenette J, Rosenblatt D, Chan T, Rideout G, Vincent R (2013). Nitrogen dioxide and ultrafine particles dominate the biological effects of inhaled diesel exhaust treated by a catalyzed diesel particulate filter. Toxicol Sci.

[66] Kobayshi T, Miyauchi T, Sakai S, Maeda S, Yamaguchi I, Goto K, Sugishita Y (1998). Down-regulation of ET(B) receptor, but not ET(A) receptor, in congestive lung secondary to heart failure. Are marked increases in circulating endothelin-1 partly attributable to decreases in lung ET(B) receptor-mediated clearance of endothelin-1?. Life Sci.

[67] Khimji AK, Rockey DC (2010). Endothelin–biology and disease. Cell Signal.

[68] Bourden JA, Saber AT, Jacobsen NR, Williams A, Vogel U, Wallin H, Hallppanavar S, Yauk CL (2013). Carbon black nanoparticle Intratracheal instillation does not alter cardiac gene expression. Cardiovasc Toxicol.

[69] Schulz H, Muhle H, Krinke GJ (2000). The laboratory Rat. The handbook of experimental animals.

[70] Singh U, Jialal I (2006). Oxidative stress and atherosclerosis. Pathophysiology.

[71] Victor VM, Rocha M, Sola E, Banuls C, Garcia-Malpartida K, Hernandez-Mijares A (2009). Oxidative stress, endothelial dysfunction and atherosclerosis. Curr Pharm Des.

[72] Li Z, Hulderman T, Salmen R, Chapman R, Leonard SS, Young SH, Shvedova A, Luster MI, Simeonova PP (2007). Cardiovascular effects of pulmonary exposure to single-wall carbon nanotubes. Environ Health Perspect.

[73] Choi AM, Alam J (1996). Heme oxygenase-1: function, regulation, and implication of a novel stress-inducible protein in oxidant-induced lung injury. Am J Respir Cell Mol Biol.

[74] Stoeger T, Reinhard C, Takenaka S, Schroeppel A, Karg E, Ritter B, Heyder J, Schulz H (2006). Instillation of six different ultrafine carbon particles indicates a surface area threshold dose for acute lung inflammation in mice. Environ Health Perspect.

[75] Brown JS, Wilson WE, Grant LD (2005). Dosimetric comparisons of particle deposition and retention in rats and humans. Inhal Toxicol.

[76] Sioutas C, Delfino RJ, Singh M (2005). Exposure assessment for atmospheric ultrafine particles (UFPs) and implications in epidemiologic research. Environ Health Perspect.

[77] Semmler M, Seitz J, Erbe F, Mayer P, Heyder J, Oberdörster G, Kreyling WG (2004). Long-term clearance kinetics of inhaled ultrafine insoluble iridium particles from the rat lung, including transient translocation into secondary organs. Inhal Toxicol.

[78] Geiser M, Kreyling WG (2010). Deposition and biokinetics of inhaled nanoparticles. Part Fibre Toxicol.

[79] Rich DQ, Ozkaynak H, Crooks J, Baxter L, Burke J, Ohman-Strickland P, Thevenet-Morrison K, Kipen HM, Zhang J, Kostis JB, Lunden M, Hodas N, Turpin BJ (2013). The triggering of myocardial infarction by fine particles is enhanced when particles are enriched in secondary species. Environ Sci Technol.

[80] Barnett AG, Williams GM, Schwartz J, Best TL, Neller AH, Petroeschevsky AL, Simpson RW (2006). The effects of air pollution on hospitalizations for cardiovascular disease in elderly people in Australian and New Zealand cities. Environ Health Perspect.

[81] Harder V, Gilmour P, Lentner B, Karg E, Takenaka S, Ziesenis A, Stampfl A, Kodavanti U, Heyder J, Schulz H (2005). Cardiovascular responses in unrestrained WKY rats to inhaled ultrafine carbon particles. Inhal Toxicol.

[82] Kodavanti UP, Moyer CF, Ledbetter AD, Schladweiler MC, Costa DL, Hauser R, Christiani DC, Nyska A (2003). Inhaled environmental combustion particles cause myocardial injury in the Wistar Kyoto rat. Toxicol Sci.

[83] Kodavanti UP, Schladweiler MC, Ledbetter AD, Watkinson WP, Campen MJ, Winsett DW, Richards JR, Crissman KM, Hatch GE, Costa DL (2000). The spontaneously hypertensive rat as a model of human cardiovascular disease: evidence of exacerbated cardiopulmonary injury and oxidative stress from inhaled emission particulate matter. Toxicol Appl Pharmacol.

[84] Karoly ED, Li Z, Dailey LA, Hyseni X, Huang YC (2007). Up-regulation of tissue factor in human pulmonary artery endothelial cells after ultrafine particle exposure. Environ Health Perspect.

[85] Franchini M, Guida A, Tufano A, Coppola A (2012). Air pollution, vascular disease and thrombosis: linking clinical data and pathogenic mechanisms. J Thromb Haemost.

[86] Kodavanti UP, Schladweiler MC, Ledbetter AD, Hauser R, Christiani DC, McGee J, Richards JR, Costa DL (2002). Temporal association between pulmonary and systemic effects of particulate matter in healthy and cardiovascular compromised rats. J Toxicol Environ Health A.

